# Prion infectivity is encoded exclusively within the structure of proteinase K-resistant fragments of synthetically generated recombinant PrP^Sc^

**DOI:** 10.1186/s40478-018-0534-0

**Published:** 2018-04-24

**Authors:** Fei Wang, Xinhe Wang, Romany Abskharon, Jiyan Ma

**Affiliations:** 1 Center for Neurodegenerative Science, Van Andel Research Institute, 333 Bostwick Avenue N.E., Grand Rapids, MI 49503 USA; 2 National Institute of Oceanography and Fisheries (NIOF), Cairo, 11516 Egypt

**Keywords:** Prion disease, Prion, PrP^Sc^, Recombinant prion, Proteinase K-resistant, Prion infectivity, Protein conformation

## Abstract

Transmissible spongiform encephalopathies, also known as prion diseases, are a group of fatal neurodegenerative disorders affecting both humans and animals. The central pathogenic event in prion disease is the misfolding of normal prion protein (PrP^C^) into the pathogenic conformer, PrP^Sc^, which self-replicates by converting PrP^C^ to more of itself. The biochemical hallmark of PrP^Sc^ is its C-terminal resistance to proteinase K (PK) digestion, which has been historically used to define PrP^Sc^ and is still the most widely used characteristic for prion detection. We used PK-resistance as a biochemical measure for the generation of recombinant prion from bacterially expressed recombinant PrP. However, the existence of both PK- resistant and -sensitive PrP^Sc^ forms in animal and human prion disease led to the question of whether the in vitro-generated recombinant prion infectivity is due to the PK-resistant or -sensitive recombinant PrP forms. In this study, we compared undigested and PK-digested recombinant prions for their infectivity using both the classical rodent bioassay and the cell-based prion infectivity assay. Similar levels of infectivity were detected in PK-digested and -undigested samples by both assays. A time course study of recombinant prion propagation showed that the increased capability to seed the conversion of endogenous PrP in cultured cells coincided with an increase of the PK-resistant form of recombinant PrP. Moreover, prion infectivity diminished when recombinant prion was subjected to an extremely harsh PK digestion. These results demonstrated that the infectivity of recombinant prion is encoded within the structure of the PK-resistant PrP fragments. This characteristic of recombinant prion, that a simple PK digestion is able to eliminate all PK-sensitive (non-infectious) PrP species, makes possible a more homogenous material that will be ideal for dissecting the molecular basis of prion infectivity.

## Introduction

Prion diseases are a group of fatal neurodegenerative disorders affecting both human and animals [20]. Prion is the causative agent of prion disease, which is a protein-conformation-based pathogen called PrP^Sc^ [19, 20]. The normal form of prion protein, PrP^C^, is a glycosylphosphatidylinositol-anchored protein widely expressed in tissues and enriched in the central nervous system [2, 20]. PrP^Sc^ propagates by coercing the conversion of PrP^C^ into PrP^Sc^, resulting in more PrP^Sc^, neurodegeneration, and the manifestation of prion disease [12]. Structural studies have shown that PrP^C^ contains a flexible N-terminal region and a fully folded C-terminal globular domain with three α-helices [22, 23]. No high-resolution structure of PrP^Sc^ is available [41], but biophysical studies have provided strong evidence that PrP^Sc^ is composed almost exclusively of β-sheeted structures [30, 33], indicating that the PrP^C^-to-PrP^Sc^ conversion involves a major structural rearrangement. Elucidating the three-dimensional structure of PrP^Sc^, the structural basis of the PrP^Sc^ seeding process, and the structural rearrangement of the PrP^C^-to-PrP^Sc^ conversion are the major goals of current prion research. This information will provide the foundation for developing effective prophylactic and therapeutic strategies against these fatal neurodegenerative disorders [3].

Prions were once considered an unorthodox disease-causing agent, but now it is clear that in other common, late-onset, neurodegenerative diseases, protein aggregates such as Aβ and tau in Alzheimer’s disease and α-synuclein in Parkinson’s disease are able to propagate their diseased conformations in a “prion-like” manner [8, 21, 31]. Among all these protein aggregates, PrP^Sc^ is the only one that has been proven to be a disease-causing agent. Elucidating the molecular mechanism of prion infectivity is not only important for combatting prion disease, but will also shed light on the molecular mechanisms of propagation of other protein aggregates.

Using bacterially expressed recombinant PrP (recPrP), we have generated recombinant prion (recPrP^Sc^) in vitro*,* which causes bona fide prion disease in wild-type animals [36–38, 40]. Similar to brain-derived PrP^Sc^, recPrP^Sc^ exhibits the signature C-terminal PK resistance, is highly infectious, and has almost all the biological properties of a native prion in diseased tissue [29, 35, 38]. However, it is still unclear whether the prion infectivity is encoded within the structure of the PK-resistant recPrP^Sc^ fragments; that is, whether the PK-resistant recPrP^Sc^ fragments contain the full prion infectivity or some of the infectivity is actually from PK-sensitive recPrP^Sc^. The answer to this question is important, because it has been demonstrated in human and animal prion diseases that both PK-resistant and PK-sensitive PrP^Sc^ forms exist and that both contribute to prion infectivity [5, 15, 24–26, 32]. Therefore, some PK-sensitive recPrP^Sc^ may be responsible for part or all of the recombinant prion infectivity.

The in vitro-generated recPrP^Sc^ provides an excellent platform for dissecting the molecular basis of prion infectivity. However, a difficulty of this type of study is the heterogeneity of the recPrP^Sc^ preparation. That is, multiple recPrP conformers are generated during the in vitro conversion process and less than 10% of recPrP becomes PK-resistant. Understanding whether the PK-resistant or PK-sensitive recPrP contributes to prion infectivity would have a huge impact on the mechanistic study of prion infectivity. If the infectivity were encoded by the PK-resistant recPrP^Sc^ fragments, then PK digestion would remove all the PK-sensitive recPrP species and greatly enhance the homogeneity. If PK-sensitive recPrP is responsible for the infectivity, more effort will need to be spent on dissecting the PK-sensitive recombinant prion fraction.

We performed a detailed study of the replication process of recPrP^Sc^ and found a clear association between the recPrP conformational change and the propagation of prion infectivity. We also compared the prion infectivity of PK-digested and -undigested recPrP^Sc^ samples using both a cell-based prion infectivity assay and a rodent bioassay. Our results clearly showed that recombinant prion infectivity was fully encoded within the PK-resistant conformation.

## Materials and methods

### Generation of recPrP^Sc^

The purification of recombinant murine PrP 23–230, the preparation of substrates, and serial protein misfolding cyclic amplification (sPMCA) were performed as previously described [34, 36, 37]. For seeded sPMCA, 10 μL of recPrP^Sc^ seed was added to the substrate and the mixture was subjected to one round of PMCA. After each round, 10 μL of the product was transferred to a new tube containing 90 μl of substrate for another round. To detect the generation of recPrP^Sc^, 10 μL of PMCA product after each round (or at designated time points within the same 24-h round) was incubated with 10 μL proteinase K (PK; 100 μg/mL unless stated otherwise) for 30 min at 37 °C, followed by the addition of 2 mM phenylmethylsulfonyl fluoride. The PK-digested samples were subjected to SDS-PAGE and western blotting. All the PK-resistant PrP fragments were detected using POM1 primary anti-PrP antibody [18].

### The enzyme-linked immunospot (Elispot) cell infection assay

The Elispot assay was adapted from previous studies [11, 13] with minor modifications. Briefly, 100 μL of samples were collected after one round of PMCA (or at designated time points within the same round). The collected samples were either untreated or treated with benzonase, PK, or both, and centrifuged at 100,000 × *g* and 4 °C for 1 h. The pellets were then washed twice with PBS and centrifuged at 100,000 × *g* and 4 °C for 1 h after each wash. After the second wash, the pellets were resuspended in 100 μL of CAD5 growth media (OPTI-MEM, 5% BGS, and 1% penicillin and streptomycin) and sonicated for 30 s using a cup-hold Misonic Sonicator (XL2020) at 50% output. Then each sample was serially diluted, and 60 μL of undiluted or diluted samples were used to infect CAD5 cells. After two 1:10 splits, 20,000 CAD5 cells/well were transferred to the Millipore 96-well Elispot plates (MSIPN4W) and subjected to the Elispot assay. The images were taken by a S6 Micro Analyzer (CTL Analyzers, LLC) and processed by the ImmunoSpot software (CTL Analyzers, LLC). The graph was generated using GraphPad Prism (GraphPad Software, Inc.). The end-point titration data was used to calculate the infectivity titer in CAD5 cells according to the Spearman-Karber formula.

### Mouse bioassays

The mouse bioassays were performed as previously described [34, 36–38, 40]. In brief, 20 μL of purified recPrP^Sc^, either untreated or treated with Benzonase, PK, or both, was inoculated into a mouse intracerebrally with or without serial dilution. The infectivity titer was calculated according to the Spearman-Karber formula.

### Ethics statement

This study was carried out in accordance with the *Guide for the Care and Use of Laboratory Animals* of the National Institutes of Health. The protocols were approved by the Institutional Animal Care and Use Committee of the Van Andel Research Institute (Assurance Number A4383–01).

## Results

### Amplification of recPrP^Sc^ in vitro was accompanied by an increase of prion infectivity

Previously, we demonstrated that recPrP^Sc^ generated through sPMCA with recPrP and two cofactors—specifically, 1-palmitoyl-2-oleoyl-*sn*-glycero-3-phospho-(1′-*rac*-glycerol) (POPG) and total RNA isolated from normal mouse liver—is highly infectious to wild-type mice and has an infectious dose 50 (ID_50_) around 10^4^/μg recPrP [36, 38]. The mouse bioassay of prion infectivity is robust and accurate, but because of the time required and the cost, it is not well suited for detailed analysis of infectivity. The Elispot cell-based assay is a new prion infectivity assay [13, 14] that is quantitative and rapid. It has been used to reveal the relationship between prion infectivity and neurotoxicity [27] and the evolution of a prion when it is exposed to changing environments [11]. Because in vitro-generated recPrP^Sc^ is able to chronically infect susceptible cultured cells [37], we adapted this assay for our study.

**Fig. 1:**
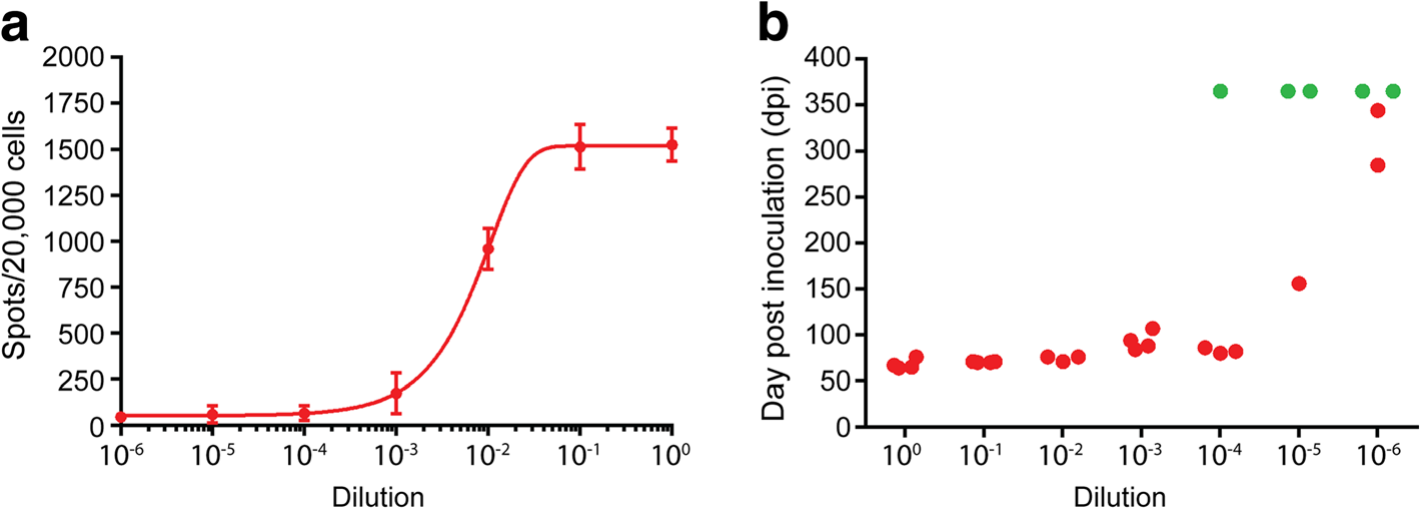
Titer of recPrP^Sc^ infectivity determined by end-point titration with the Elispot cell infection assay (**a**) and the tga20 mouse bioassay (**b**). The same batch of recPrP^Sc^ was subjected to 10-fold serial dilution, and both undiluted and diluted recPrP^Sc^ were used to infect naïve CAD5 cells or to intracerebrally inoculate tga20 mice. In **b**, red dots indicate mice that reached the terminal stage of disease; green dots represent mice without any disease at the end of 365 dpi

We compared the infectious titer of recPrP^Sc^ in CAD5 cells by the Elispot cell infection assay with the titer in PrP-overexpressing tga20 transgenic mice by bioassay [7]. The same batch of recPrP^Sc^ was used for both assays (Fig. 1) and ID_50_s were calculated following standard methods [14]. We found that the ID_50_ of recPrP^Sc^ obtained from CAD5 cells was 3.33 × 10^5^/μg recPrP and from tga 20 mice was 2.00 × 10^5^/μg recPrP, indicating that the Elispot infection assay can be as sensitive as the bioassay. Although it is well known that prion infection in cultured cells can be influenced by the prion strain [13], the similar titers suggest that, at least for recPrP^Sc^ generated with our procedure, the Elispot assay can be used to track changes of infectivity. Thus, we used this assay to analyze the relationship between prion infectivity and the PK-resistant recPrP^Sc^ conformation.

**Fig. 2:**
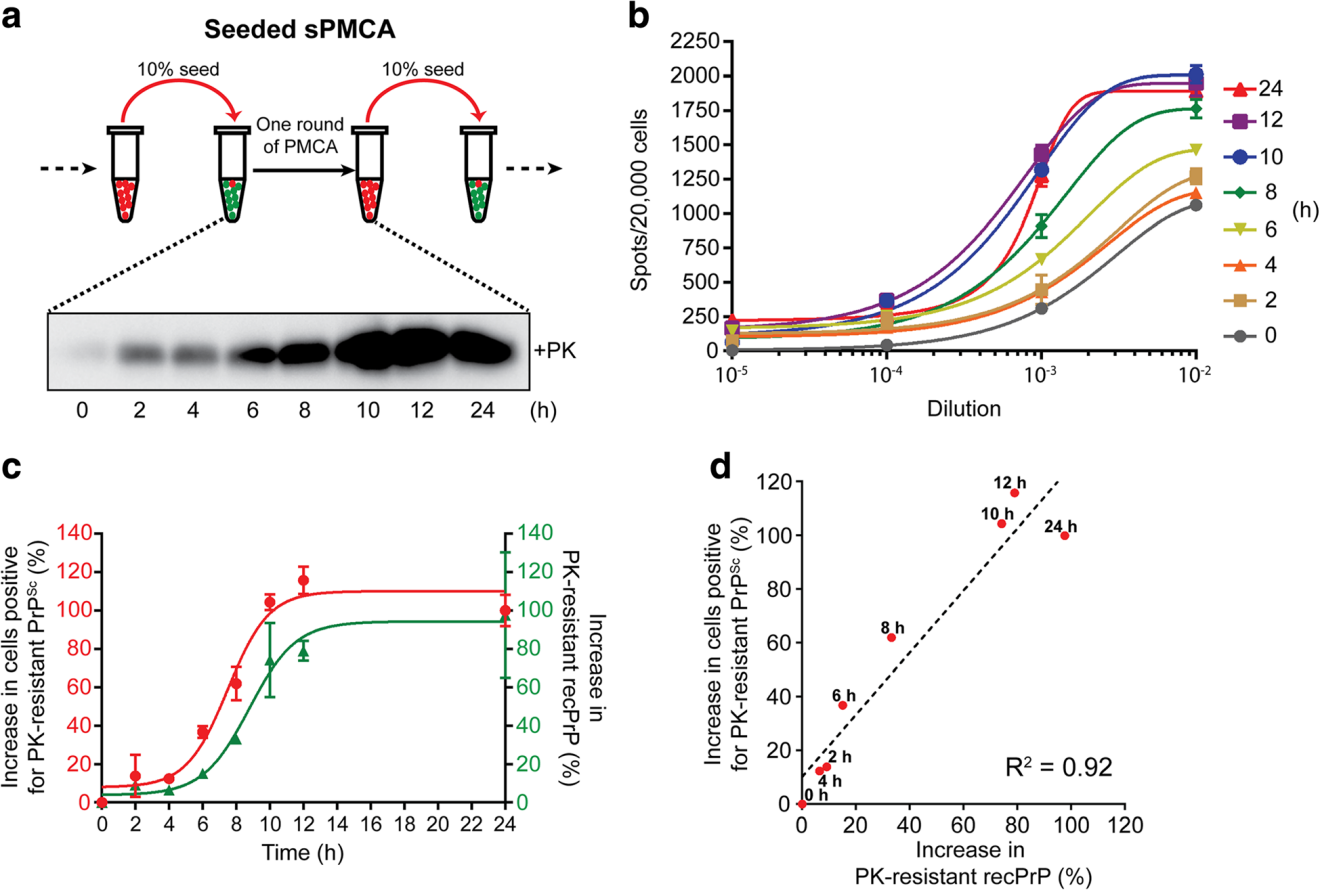
Prion infectivity and the amount of PK-resistant recPrP conformer increased concurrently. **a** Upper panel: an illustration of recPrP^Sc^ amplification in sPMCA. Lower panel: detection of PK-resistant recPrP at 0, 2, 4, 6, 8, 10, 12, and 24 h within a single round. **b** The infectivity of sPMCA products collected in (**a**) was evaluated by Elispot CAD5 cell infection assay. **c** and **d** The amplification of the PK-resistant recPrP conformation correlated with prion infectivity. The percentage increase of cells positive for PK-resistant PrP^Sc^ (red dots, left Y axis) and of PK-resistant recPrP^Sc^ (green triangles, right Y axis) within a single round of sPMCA were plotted as a function of time (h) (**c**). The percentage increase of cells positive for PK-resistant PrP^Sc^ was plotted versus the percentage increase of PK-resistant recPrP^Sc^ (**d**). The linear regression analysis was performed and a linear regression line (dashed line) was added to the plot (Equation: Y = 1.156*X + 10.19, R^2^: 0.92)

We first asked whether prion infectivity correlates with the increase of PK-resistant PrP during the sPMCA. Previous studies have shown that the end product of sPMCA is infectious [36–38], but we still don’t know the details of how prion infectivity changes during the amplification process. Each round of sPMCA in our protocol consists of 48 cycles of sonication (30 s) and incubation (29 min and 30 s). We collected samples at various time points during one round of PMCA (Fig. 2a), which showed that the proportion of PK-resistant recPrP increased gradually in the first 12 h and remained relatively unchanged in the second 12 h. When the same set of samples was used to infect CAD5 cells, a time-dependent increase of prion infectivity was obvious and, notably, was mainly increased during the first 12 h (Fig. 2b). Comparing the infectivity at 10^− 3^ dilution with the change in the PK resistance of recPrP produced a good correlation (Fig. 2c and d). These results suggest that the increase of prion infectivity correlated with the increase of PK-resistant recPrP during sPMCA, strongly supporting the idea that the conformational change of recPrP is responsible for the generation of prion infectivity during sPMCA.

### Prion infectivity is encoded within the PK-resistant recPrP^Sc^ fragments

**Fig. 3:**
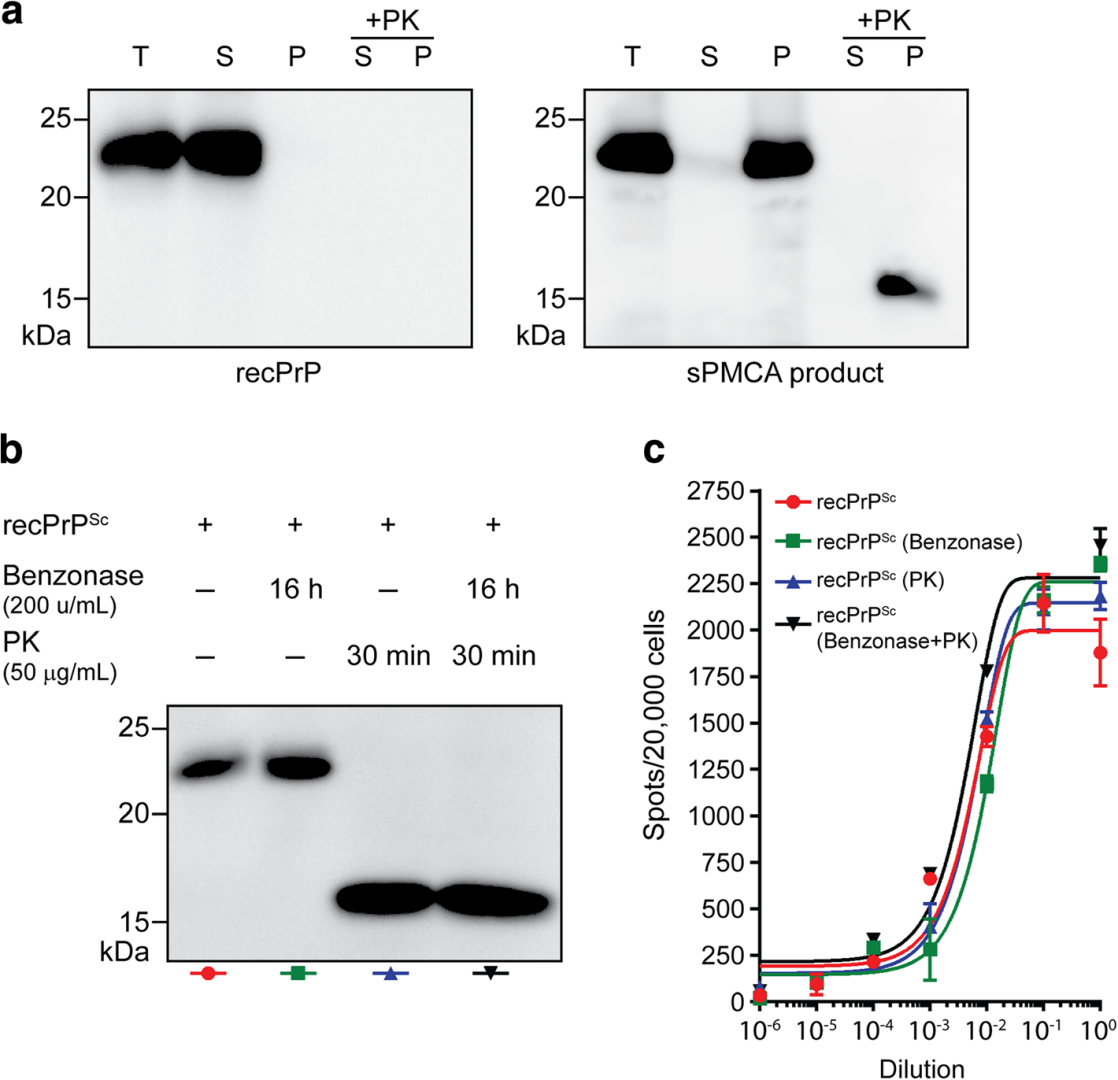
PK digestion did not reduce prion infectivity. **a** RecPrP (left panel) and the sPMCA product (right panel) were subjected to ultracentrifugation (100,000 × *g*, 1 h, 4 °C) with or without prior PK digestion (50 μg/mL, 30 min, 37 °C) as indicated (T: total input, 1 μL; S: supernatant fraction, 1 μL; P: pellet fraction, 1 μL). For PK digestion, 10 μL of sample from the supernatant or pellet fraction was used. **b** The same batch of recPrP^Sc^ was untreated or was treated with Benzonase, PK, or both, as indicated. **c** Prion infectivity of samples in (**b**) was evaluated by Elispot cell infection assay

RecPrP without any treatment is soluble (Fig. 3a, left), but almost all recPrP became insoluble after sPMCA, with a large portion of the insoluble recPrP remaining PK-sensitive (Fig. 3a, right). To determine whether prion infectivity is associated with the PK-resistant recPrP^Sc^, we subject aliquots from the same batch of recPrP^Sc^ preparation to Benzonase treatment (removing free RNA), PK digestion (removing PK-sensitive recPrP), or both, and compared their infectivity with that of the untreated aliquot. Benzonase digestion alone did not cause noticeable change in total recPrP (Fig. 3b). PK digestions with or without Benzonase generated similar amounts of PK-resistant recPrP^Sc^, in which PK-sensitive recPrP and the N-terminus of recPrP^Sc^ were removed (Fig. 3b). When these treated aliquots were compared with untreated aliquot for infectivity in CAD5 cells, we found that none of the treatments appeared to alter infectivity (Fig. 3c). Because all the sPMCA products were in detergent solution and the pelleting step before cell infection would remove all free phospholipid POPG, this result suggests that the PK-resistant recPrP^Sc^ aggregate contained all the prion infectivity. This conclusion was verified by bioassay in tga20 mice, which showed that the PK digestion and Benzonase treatment did not reduce the infectivity of the PMCA product. The results of mouse bioassays, which were performed with different batches of PMCA products, are summarized in Table 1.

**Table 1:** Bioassay in tga20 mice via intracerebral inoculation^a^

Experiment	Inoculum		Attack rate (*N*_diseased_/*N*_total_)	Survival time for individual mice (dpi)^d^
#1	recPrP^Sc^	Cofactors for #1-#3: • POPG • Total mouse liver RNA	100% (4/4)	85, 86, 98, 121
#2	recPrP^Sc^		100% (3/3)	91, 93, 94
#3	recPrP^Sc^ (after Benzonase+PK digestion)		100% (5/5)	73, 76, 76, 80, 80
#4	recPrP^Sc^	Cofactors for #4-#6: • POPG • Synthetic Poly rA RNA^c^	100% (4/4)	104, 109, 115, 119
#5 ^b^	recPrP^Sc^		100% (4/4)	95, 95, 104, 110
#6 ^b^	recPrP^Sc^ (after Benzonase+PK digestion)		100% (4/4)	95, 106, 108, 109

^a^Different batches of recPrP^Sc^ were used

^b^The same batch of recPrP^Sc^ was used to for these two experiments

^c^See reference [37]

^d^dpi: days post injection

**Fig. 4:**
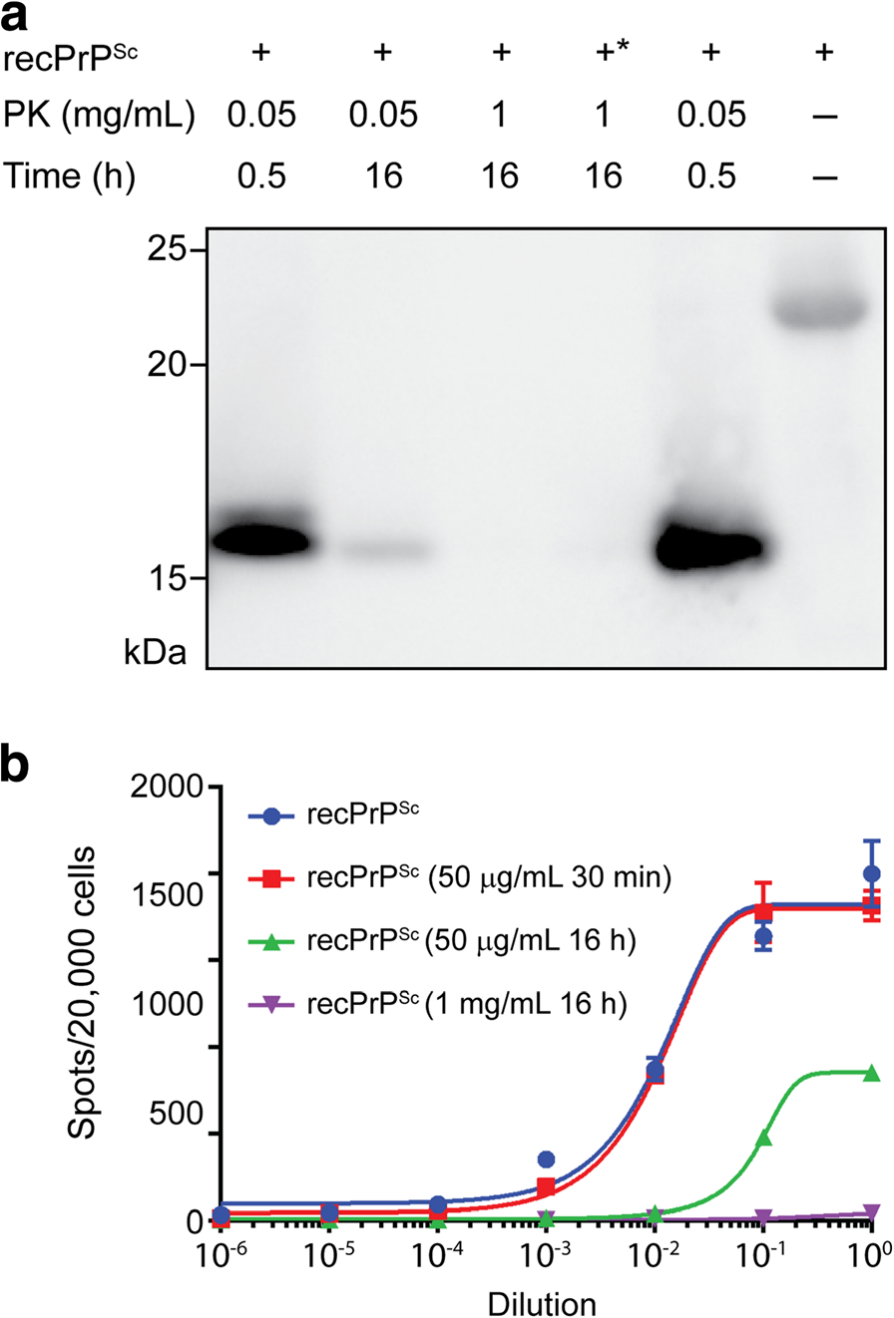
The recombinant prion infectivity is encoded within its PK-resistant protein conformation. **a** Samples from the same batch of recPrP^Sc^ were treated with PK as indicated. The lane marked by * was loaded with 5 times of PK-resistant recPrP^Sc^ (1 mg/mL, 37 °C, 16 h). **b** The prion infectivity of samples in (**a**) as evaluated by the Elispot cell infection assay

PrP^Sc^ can be almost completely digested by extended treatment with PK at high concentration [4]. We took advantage of this property and performed a harsh PK digestion to determine whether the disappearance of the PK-resistant recPrP^Sc^ correlated with a reduction in prion infectivity. Relative to that of an untreated sample, our standard PK digestion (50 μg/mL, 37 °C, 30 min) did not cause any change in the infectivity (Fig. 4a). An extended digestion (50 μg/mL, 37 °C, 16 h) not only resulted in much less PK-resistant recPrP^Sc^ (Fig. 4a) but also dramatically decreased the infectivity (Fig. 4b). When recPrP^Sc^ was subjected to an extreme PK digestion (1 mg/mL, 37 °C, 16 h), the PK-resistant recPrP band was barely detectable, and concomitantly the prion infectivity was virtually eliminated (Fig. 4b). These results provided further evidence supporting the correlation of prion infectivity to the conformation of PK-resistant recPrP^Sc^.

## Discussion

In this study, we dissected the propagation of recombinant prion and established a clear correlation between the amplification of the PK-resistant recPrP^Sc^ conformation and the increase of prion infectivity. Moreover, our findings demonstrated that the recombinant prion infectivity is encoded within the structure of its PK-resistant fragments, confirming that the self-propagating, PK-resistant recPrP^Sc^ is the infectious entity.

Prion infectivity is classically determined by an endpoint dilution assay in animals [9], which is accurate but time consuming (> 1 year) and costly. That is why it is not used for detailed studies of prion infectivity. Weissmann and colleagues developed the new Elispot cell-based prion infection assay [10, 11, 13, 14]. Relative to the traditional animal bioassay, it is rapid, cost-effective, and more suitable for detailed characterization of prion infectivity. The Elispot assay has the drawback that only a few cell lines are susceptible to prion infection, and those cell lines have shown different sensitivities to various prion strains [13]. Among the susceptible cell lines, CAD5 cells have a high susceptibility to prion infection and a high sensitivity to almost all murine prion strains tested [11, 13]. Thus, we selected CAD5 cells to test our in vitro–generated recPrP^Sc^. The similar levels of infectivity found by the CAD5 cell infection assay and the classic tga20 mouse bioassay support that idea the cell assay is well suited for measuring the infectivity of the recPrP^Sc^ generated by our protocol.

The CAD5 cells and the Elispot assay allowed us to perform a detailed characterization of the relationship between PK resistance and prion infectivity. Although PrP^Sc^ has been traditionally defined as the PK-resistant PrP species, the so-called PK resistance reflects its higher tolerance to PK digestion relative to normal PrP^C^. However, the generally accepted PK digestion conditions are chosen for practicality, which ensure the removal of all normal PrP^C^ every time but may degrade some PrP^Sc^ species as well. Using the standard PK digestion conditions, several groups have found PK-sensitive PrP^Sc^ with various methods [5, 15, 17, 24–26, 28, 32]. Cronier et al. reported that a large proportion of the Rocky Mountain Laboratory (RML) murine prion strain consists of at least 2 types of PK-sensitive PrP^Sc^: one is thermolysin-resistant and contributes little to prion infectivity, while the other is thermolysin-sensitive and makes up of about 80% of the RML infectious titer as measured by the cell-based assay [5]. A more recent study by Sajnani et al. showed that the PK-sensitive PrP^Sc^ isolated from diseased brains by ultracentrifugation is also infectious, and its infectivity (measured by animal bioassay) is comparable to that of the PK-resistant PrP^Sc^ [26]. More importantly, they demonstrated that the PK-sensitive and PK-resistant PrP^Sc^ share common structural features [26], indicating that the basic units of these two types of PrP^Sc^ aggregate share the same protein conformation. Thus, the difference in PK sensitivity could be a result of the degree of multimerization, binding to other molecules, or both.

An interesting finding of our study is that the prion infectivity was encoded almost exclusively by the PK-resistant recPrP conformation, which suggests that compared to PrP^Sc^ from diseased tissues that consists of both PK-resistant and -sensitive PrP forms, the recPrP^Sc^ is relatively more homogeneous with recPrP species of higher PK-resistance. The simplest explanation would be that the in vitro–generated recPrP^Sc^ might be structurally different from PrP^Sc^ from diseased tissue, which could be a result of the lack of post-translational modification or of a difference in PrP^Sc^ structure. This is unlikely, however, because the biological activity and the structural characteristics of recPrP^Sc^ are almost identical to those of PrP^Sc^ [35, 38], and a recent limited-proteolysis study revealed a shared structure between recPrP^Sc^ and brain-derived PrP^Sc^ [29]. A more plausible explanation could be the simplicity of the in vitro recPrP conversion system. Besides recPrP, only Triton X-100 and two cofactors, POPG and RNA, are present, which is different from the multitude of molecules present in tissue lysates. As a result, the converted recPrP^Sc^ is less likely to bind to other molecules to maintain the oligomeric or the less PK-resistant state. Instead, in the absence of other molecules, recPrP^Sc^ is probably more prone to multimerization to form aggregates with higher PK-resistance. Consistent with this idea, Noble et al. reported that in their recPrP^Sc^ preparation system that uses only one cofactor, around 90% of recPrP was converted into the PK-resistant form [16]. Although it remains unclear how much of infectivity the 10% PK-sensitive recPrP may account for, the higher conversion rate in their system supports the notion that less cofactor may result in more aggregated and PK-resistant recPrP. It is also worth noting that our recPrP^Sc^ and the recPrP^Sc^ generated by Noble et al. appear to be two different prion strains [6]. It will be of high interest to compare their structural differences in the future.

The fact that infectivity is exclusively found in the PK-resistant recPrP^Sc^ species offers a great advantage in using this material to study the molecular basis of prion infectivity, which is one of the most important applications of the in vitro conversion system. The aggregated nature of PrP^Sc^ in diseased tissues limits the possibility of applying conventional structural biology tools [39]. It requires the integration of multiple biochemical and biophysical techniques to piece together the high-resolution PrP^Sc^ structure, which demands a reliable source of prions [29, 39]. As mentioned above, the recPrP^Sc^ generated in our system recapitulates all the properties of native prions and is a convenient and robust source of PrP^Sc^ [1, 29, 36–38]. Because infectivity is associated with PK-resistant recPrP^Sc^, we will be able to remove all PK-sensitive (non-infectious) recPrP species by a simple proteinase K digestion. The increased homogeneity will greatly facilitate structural studies of recPrP^Sc^ and studies to identify potential therapeutic agents that are able to neutralize prion infectivity.

## Conclusions

In conclusion, our results clearly establish that the structure of the PK-resistant recPrP^Sc^ fragments contains all the information for prion infectivity and there is no infectious PK-sensitive recPrP^Sc^ in the synthetically generated prion prepared with our protocol. Moreover, we also show that the increase of the PK-resistant recPrP conformation during PMCA correlates with an increase of prion infectivity, supporting that recPrP^Sc^ is indeed a protein conformation based infectious agent.
